# Antibody Responses to Natural SARS-CoV-2 Infection or after COVID-19 Vaccination

**DOI:** 10.3390/vaccines9080910

**Published:** 2021-08-16

**Authors:** Haya Altawalah

**Affiliations:** 1 Department of Microbiology, Faculty of Medicine, Kuwait University, Safat 24923, Kuwait; haya.altawalah@ku.edu.kw or dr.altawalah@gmail.com; 2 Virology Unit, Yacoub Behbehani Center, Sabah Hospital, Ministry of Health, Safat 24923, Kuwait

**Keywords:** COVID-19, humoral immunity, neutralizing antibody, SARS-CoV-2, serology assays

## Abstract

The novel coronavirus, severe acute respiratory syndrome coronavirus 2 (SARS-CoV-2), is the causative agent of the ongoing pandemic of coronavirus disease 2019 (COVID-19). The clinical severity of COVID-19 ranges from asymptomatic to critical disease and, eventually, death in smaller subsets of patients. The first case of COVID-19 was declared at the end of 2019 and it has since spread worldwide and remained a challenge in 2021, with the emergence of variants of concern. In fact, new concerns were the still unclear situation of SARS-CoV-2 immunity during the ongoing pandemic and progress with vaccination. If maintained at sufficiently high levels, the immune response could effectively block reinfection, which might confer long-lived protection. Understanding the protective capacity and the duration of humoral immunity during SARS-CoV-2 infection or after vaccination is critical for managing the pandemic and would also provide more evidence about the efficacy of SARS-CoV-2 vaccines. However, the exact features of antibody responses that govern SARS-CoV-2 infection or after vaccination remain unclear. This review summarizes the main knowledge that we have about the humoral immune response during COVID-19 disease or after vaccination. Such knowledge should help to optimize vaccination strategies and public health decisions.

## 1. Introduction

Severe acute respiratory syndrome coronavirus 2 (SARS-CoV-2) can cause asymptomatic infection, mild-to-moderate, and severe manifestations such as acute respiratory distress syndrome (ARDS), vascular and neurological complications, and eventually death [1,2,3]. From the end of 2019, SARS-CoV-2 agent has emerged, with devastating consequences for humankind. Thus, until now, COVID-19 still has a significant toll globally, claiming roughly 4 million lives and negatively affecting the global economy.

SARS-CoV-2, a member of the *Betacoronavirus* genus within the *Nidovirales* order, consists of a nucleocapsid (N) protein surrounded by an envelope containing three membrane proteins: spike (S)—divided into two functional subunits, S1 and S2, membrane (M), and envelope (E) proteins [4]. Among the four coronavirus structural proteins, S and N proteins are the main immunogens [5,6]. SARS-CoV-2 uses angiotensin-converting enzyme 2 (ACE2) as a receptor, which is bound by the receptor-binding domain (RBD) on the S1 subunit [4,7,8]. Moreover, transmembrane protease serine 2 (TMPRSS2) and cathepsin B/L are also involved in viral entry [9]. The RBD is highly immunogenic and elicits antibodies that are strongly correlated with SARS-CoV-2 neutralization [10]. Therefore, neutralizing the RBD is fundamental to block the pathogenesis of SARS-CoV-2 infection [11,12,13].

SARS-CoV-2 infection produces early detectable humoral immune responses in most patients. In addition, SARS-CoV-2 leads to the development of neutralizing antibodies (NAbs) in the vast majority of cases [14,15,16,17]. However, the duration and the protective capacity of the humoral immune response are still unknown. Several studies have shown the appearance of neutralizing and protective anti-SARS-CoV-2 antibodies after infection, which reduced the risk of reinfection in the following 13 months [18].

Herd immunity is critical for long-term control of the SARS-CoV-2 [19]. COVID-19 vaccination was suggested as the only viable path to reach herd immunity rather than by natural infection [20,21]. In fact, the variables that may affect the establishment of effective herd immunity are vaccine efficacy, longevity of the immunity (i.e., neutralization antibody and memory cells), and the potential emergence of variants [19]. Within short times, great investigation efforts have led to the development of effective vaccines and authorized for emergency use [22]. Furthermore, vaccine studies are conducted on neutralizing antibodies and may play an important role in controlling viral infection [23,24,25,26]. Hence, functional and durable immunity against SARS-CoV-2 are currently under investigation, as plasma antibody levels have been shown to decrease during recovery.

There have been considerable efforts to understand the immune response during SARS-CoV-2 infection and after vaccination. Reinfections have been reported with three of the four seasonal human coronaviruses (HCoVs) (HCoV-229E, -OC43, -NL63, and -HKU1) [27]. Reinfection after documented infection has been well documented in patients with SARS-CoV-2 [28,29,30,31,32,33]. Understanding the risk of reinfection with SARS-CoV-2 provides an avenue to understand the path to protection against COVID-19 [34].

The future will depend heavily on the type of immunity acquired through infection or vaccination and how the virus evolves [35]. Currently, variants of SARS-CoV-2 are emerging as an important key in determining whether COVID-19 vaccines could be sufficiently effective against these new variants. Therefore, a better understanding of the type and longevity of immune responses following viral infections or after COVID-19 vaccination are critical to reveal immune mechanisms involved in protection from reinfection and protection by vaccination. Interestingly, the dynamics and persistence of immune responses in individuals infected with SARS-CoV-2 and after COVID-19 vaccination are currently under investigation. Here, we review the relevant literature to summarize the latest understanding of humoral response to SARS-CoV-2 infection or after COVID-19 vaccination.

## 2. Humoral Immune Response during SARS-CoV-2 Infection

Immunity to the seasonal coronavirus appears to last around one year, and reinfection with the same CoV frequently occurred at 12 months after infection [36]. Previous surveys of severe acute respiratory syndrome (SARS) and Middle East respiratory syndrome (MERS) have shown that antibodies were detectable in 80–100% of patients two weeks after the onset of symptoms [37,38,39,40,41]. MERS and SARS antibody levels are dramatically reduced 2–3 years after symptom onset [42,43].

Viruses elicit a broad spectrum of antiviral antibody (Ab) responses [44]. In general, there are two fundamentally different types of antiviral Ab response, NAbs, and non-neutralizing antibodies [44].

Previous reports demonstrate that people infected with SARS-CoV-2 rarely develop specific antibodies (Abs) within the first 7 days after onset of symptoms [45,46,47,48]. By 10–11 days after onset of symptoms, greater than 90% of SARS-CoV-2 patients develop specific immunoglobulin M (IgM) and immunoglobulin G (IgG) [45,46,47]. Within 17 to 19 days of symptom onset, 100% of patients tested positive for virus-specific IgG, while the fraction of patients with virus-specific IgM peaked at 94.1%, approximately 20 to 22 days after symptom onset [49]. In addition, IgM showed a slight decrease in the less than 3 weeks that followed [49]. Furthermore, the immunoglobulin A (IgA) response increased since 6–8 days, peaks at 20–22 days, and is stronger and more persistent than the IgM response [50]. Notably, IgG and IgM titers were higher in COVID-19 severe patients compared to non-severe [49,51]. Moreover, serum concentrations of specific IgA decrease one month after symptom onset but contribute to virus neutralization in saliva for a longer period of time (days 49 to 73 after symptoms) [52].

The kinetics of the immune response, its magnitude, and its relationship to disease severity during SARS-CoV-2 infection have been extensively documented. Moreover, higher titers of anti-S1 and anti-N IgG and IgM positively correlate with age and the level of lactate dehydrogenase [53]. Notably, longitudinal studies have demonstrated that neutralizing antibody responses are more robust and are associated with severe clinical manifestations [54,55,56]. Asymptomatic COVID-19 patients have a weaker immune response and faster and greater reduction of IgG titer [49], whereas Ab titers vary greatly in different patients independently of the clinical course of SARS-CoV-2 infection, and about 5% of patients have undetectable antibody titers despite confirmed infection [57].

Several longitudinal investigations have shown that most patients had detectable SARS-CoV-2 antibody responses up to 13 months after infection, giving hope that it could even last longer than predicted [18,19,58,59]. Notably, a large study of individuals with COVID-19 suggests that their neutralizing antibody levels begin to decline after roughly six to eight months [60]. Furthermore, 24% of convalescent donors at 6–8 months from initial symptoms of COVID-19 lost NAbs [60]. Nevertheless, their bodies produce memory B cells, which can produce antibodies if they are reinfected, and T cells, which can eliminate virus-infected cells [35]. Therefore, the decline of antibody levels does not negate the protective potential because of the importance of cellular responses against SARS-CoV-2 infection [15,60,61,62].

## 3. Humoral Immune Response after COVID-19 Vaccination

Worldwide, a dozen vaccines (Pfizer-BioNTech (mRNA BNT 162b2, Comirnaty, Pfizer, Groton, CT, USA), Moderna (mRNA-1273, SpikeVax, Moderna, BioNTech, Cambridge, MA, USA), AstraZeneca (AZD1222.ChAdOx1 nCoV-19, Vaxzevria, Oxford University—AstraZeneca, Oxford, UK), Johnson & Johnson (Ad26.COV2.S, Janssen Biotech, Inc., Boston, MA, USA), Sputnik V (Gam-COVID-Vac, N F Gamaleya National Research Centre for Epidemiology and Microbiology, Moscow, Russia), BBIBP-CorV (Sinopharm, the Beijing Institute of Biological Products, Beijing, China), CoronaVac (Sinovac, Sinovac Research and Development Co, Beijing, China), EpiVacCorona (Vector center of Virology, Novosibirsk, Russia), Convidicea (Ad5-nCoV, CanSino Biologics, Tianjin, China), and Covaxin (Bharat Biotech, Hyderabad, India) are currently approved for emergency use by different authorities (i.e., US Food and Drug Administration (FDA), National Medical Products Administration (NMPA), European Medicines Agency (EMA), Ministry of Health of the Russian Federation, etc.) in shorter time than what is usually observed for previous approved vaccines. Furthermore, using several platforms, over than 200 candidate vaccines are at various developmental stages [22,63]. The platforms can be divided into inactivated or live-virus vaccines, recombinant protein vaccines and vectored vaccines, and platforms using mRNA and DNA vaccines [64]. The vaccines were derived from the first clinical isolate of the Wuhan strain (NC_045512). Currently, the World Health Organization (WHO) has approved six types of vaccines (BNT162b2, mRNA-1273, ChAdOx1 nCoV-19, Ad26.COV2.S, BBIBP-CorV, and CoronaVac) [65]. Moreover, BNT162b2, mRNA-1273, AZD1222, Ad26.COV2.S, and CoronaVac) are already being assessed in phase IV clinical trials—pharmacovigilance investigations conducted by local and international institutions—[66]. These vaccines are developed using various platforms, such as mRNA to specific SARS-CoV-2 antigens; viral-vector-based, or inactivated-virus-based vaccines, and the most routes of administration are intramuscular [63,67]. To date, all the vaccines authorized for administration to the general population have shown seroconversion [66].

### 3.1. Humoral Response of mRNA-Based Vaccines

The mRNA vaccine platform is more attractive because of its rapid and low-cost manufacturing process and also has advantages as a pandemic-response strategy, given its flexibility and efficiency in immunogen design [68,69]. Safety and efficacy data from the clinical trials of the BNT162b2 (Pfizer/BioNTech vaccine), a nucleoside-modified mRNA formulated in lipid nanoparticles that encodes the SARS-CoV-2 S stabilized in its prefusion conformation, showed 95% effectivity in preventing COVID-19 and an efficacy between 90% and 100%, with very few adverse events, such as pain at the injection site, fatigue, or headaches [23,70]. In particular, BNT162b2 elicited strong antibody responses; one week after the second dose, the geometric mean of the 50% neutralization titers of SARS-CoV-2 serum was up to 3.3-fold higher than that observed in samples from individuals who recovered from COVID-19 and also showed poly-specific cellular immunity [71]. In contrast, most patients on maintenance hemodialysis developed a substantial humoral response after administration of the BNT162b2 vaccine and it was significantly lower than in the control [72], whereas 86% of patients with inflammatory rheumatic diseases developed a humoral response following the administration of the second dose of the BNT162b2 vaccine [73].

The mRNA-1273 vaccine (Moderna)—a lipid nanoparticle-encapsulated mRNA-based vaccine that encodes the prefusion stabilized full-length S protein of the SARS-CoV-2—was developed by Moderna and the Vaccine Research Center at the National Institute of Allergy and Infectious Diseases (NIAID) [74,75]. From the phase I clinical trial, the mRNA-1273 vaccine induced anti-SARS-CoV-2 immune responses in all participants within 2 weeks after the first vaccination, and the neutralizing antibody titers induced by the two-dose schedule were similar to those found in convalescent serum specimens [76]. In addition, the mRNA-1273 vaccine showed 94.1% efficacy in preventing COVID-19 disease, including severe disease with rare serious adverse events in the phase III trial [74]. Moreover, in peritoneal dialysis patients after two doses of mRNA-1273 vaccine, 97% of patients have detectable IgG antibodies to the RBD of the S1 spike antigen [77]. However, previous studies have reported a suboptimal humoral response after vaccination with mRNA-1273 and BNT162b2 in solid organ transplant recipients treated with long-term immunosuppression compared with the general population [68,78,79], whereas administration of a third dose of the BNT162b2 vaccine significantly improved the humoral response among solid-organ transplant recipients [80].

### 3.2. Humoral Response of Adenovirus-Based Vaccines

The adenovirus-based vaccine is a strategy based on adenovirus vectors, which are nonreplicating and carry a codon-optimized gene encoding the full-length SARS-CoV-2 S protein, which can boost the immune response without the need for adjuvants [69,81]

The AZD1222 (ChAdOx1 nCoV-19) consists of the replication-deficient chimpanzee adenovirus-vectored vaccine, expressing the full-length SARS-CoV-2 S protein with a tissue plasminogen activator leader sequence [82]. In an animal model, a single vaccination with ChAdOx1 nCoV-19 elicited humoral and cellular immune responses and prevented SARS-CoV-2 pneumonia [83]. Furthermore, data derived from phase I/II show that immunization with ChAdOx1 nCoV-19 provokes rapid production of antibodies against SARS-CoV-2 spike protein that peaked by day 28 and elicited the neutralizing antibody in all participants after a booster dose [82,84]. In addition, for a pooled analysis of ChAdOx1 nCoV-19 vaccine efficacy in the United Kingdom, Brazil, and South Africa, conducted before the variants emerged, an overall vaccine efficacy of 66.7% was reported [26].

In immunocompromised patients, data showed that the ChAdOx1 nCoV-19 vaccine given as prime-boost dosing given 4–6 weeks apart produced equivalent immune responses in people living with human immunodeficiency virus who are well controlled on antiretroviral therapy [85].

Ad26.COV2.S is a recombinant replication-incompetent adenovirus serotype 26 (Ad26) vector encoding a full-length and stabilized SARS-CoV-2 spike protein [86,87,88]. In phase I–IIa clinical trial involving healthy adults, a single dose of Ad26.COV2.S triggered a strong humoral response in the majority of vaccine recipients, with the presence of S-binding and -neutralizing antibodies in more than 90% of participants, regardless of age group or vaccine dose [87]. Interestingly, binding and NAbs were detected by day 57 in 100% of vaccine recipients after a single immunization [89]. In a phase III trial, a single dose of Ad26.COV2.S showed 67% efficacy but 85% efficacy in preventing against severe-critical disease, including hospitalization and death with onset at least 28 days after administration and similar safety in other phase III trials of COVID-19 vaccines [90].

Gam-COVID-Vac (Sputnik V) is a combined vector vaccine based on recombinant adenovirus type 26 (rAd26) and recombinant adenovirus type 5 (rAd5), both of which carry the gene for SARS-CoV-2 full-length glycoprotein S (rAd26-S and rAd5-S) [91]. Both components were developed, manufactured, and stored by N F Gamaleya National Research Centre for Epidemiology and Microbiology (Moscow, Russia) [91]. During the phase I trial (administration of either rAd26-S or rAd5-S alone), SARS-CoV-2 RBD-specific IgGs were detected at day 14 in 88.9% of participants after administration of rAd26-S and in 84.2% of participants after administration of rAd5-S [91]. Notably, from day 21, Sputnik V induces a strong humoral response and SARS-CoV-2 RBD-specific IgGs were detected in 100% of vaccinated participants [91]. During phase II, SARS-CoV-2 RBD-specific IgGs were detected in 85% of participants on day 14 (after priming with rAd26-S) and in 100% of participants from day 21 [91]. In addition, volunteers who received the heterologous rAd26 and rAd5 vaccine elicited the same titer of SARS-CoV-2 neutralizing antibodies as did people who had recovered from COVID-19 [91]. In the phase III trial, Sputnik V showed 91.6% vaccine efficacy with systemic and local reactions [92].

### 3.3. Humoral Response of Inactivated Virus Vaccines

Inactivated virus vaccines are a traditional method of manufacturing vaccines that are purified from virally infected cells. Usually, they are made by exposing virulent viruses to chemical or physical agents, for example, formalin or β-propiolactone, in order to knock out infectivity while maintaining immunogenicity [93]. The BBIBP-CorV (Sinopharm) is an inactivated SARS-CoV-2 vaccine candidate developed by the Beijing Institute of Biological Products (Beijing, China) containing aluminum as an adjuvant [94]. This candidate vaccine induced high levels of neutralizing antibody in rats, mice, guinea pigs, rabbits, cynomolgus monkeys, and rhesus macaques, protecting them against SARS-CoV-2 infection [94]. For example, in mice, the NAb levels at 7, 14, and 21 days in the low- (2 μg/dose) and middle-dose (4 μg/dose) groups showed significant variation, whereas no significant variation between 21 and 28 days was noted. In the high-dose group (8 μg/dose), a significant variation only was observed between 7 and 14 days [94].

In the interim report of the phase I and II trials of Sinopharm vaccine, seroconversion was noted in all participants (100%) receiving vaccines. Most participants began to produce antibody responses after the second injection, which remained at high levels 14 days after the third injection [95]. Moreover, the neutralizing antibody response was monitored for 14 days after the injections and suggested that the inactivated vaccine could effectively induce antibody production [95]. The most common adverse reaction was injection site pain, followed by fever, which were mild and self-limiting [95]. Furthermore, the BBIBP-CorV vaccine, given in two doses, was safe and well tolerated, and humoral responses against SARS-CoV-2 were induced in all vaccinees by day 42 (100% of vaccinees) [96]. In phase II, BBIBP-CorV vaccine during two-dose immunization with 4 μg of vaccine on days 0 and 21 or on days 0 and 28 resulted in higher neutralizing antibody titers than the single 8 μg dose or the 4 μg dose on days 0 and 14 [96]. In fact, the neutralizing antibody geometric mean titers on day 28 were significantly greater in the 4 μg day 0 and 14 (169.5), day 0 and 21 (282.7), and day 0 and 28 (218) schedules than the 8 μg day 0 schedule (14.7, all *p* < 0.001) [96].

CoronaVac (Sinovac Research and Development Co., Beijing, China) is an inactivated virus alum-adjuvanted candidate vaccine [97]. In the phase I/II clinical trial, seroconversion rates ranged from 92% to 100% after two doses of CoronaVac (3 μg and 6 μg, respectively) in adults aged 60 years and older [98]. Notably, the neutralizing antibody titers induced by the 3 μg dose were similar to those of the 6 μg dose and higher than those of the 1.5 μg dose [98]. Furthermore, CoronaVac was well tolerated with very few adverse events [98].

## 4. SARS-CoV-2 Variants and Humoral Immune Response

Currently, SARS-CoV-2 variants are emerging as an important key in determining whether SARS-CoV-2 infection or COVID-19 vaccines can establish sufficient herd immunity [19]. Thus, the emergence of SARS-CoV-2 variants harboring mutations in the S protein has raised concern about potential immune escape. In fact, the WHO designated Alpha (B.1.1.7), Beta (B.1.351), Gamma (P.1), and Delta (B.1.617.2) as variants of concern (VOCs) due to evidence of increased transmissibility. The ongoing evolution of SARS-CoV-2 requires continuous monitoring of the significance of changes to the humoral immune response.

The possibilities that variants may reduce the effectiveness of the vaccine are being highlighted [99]. Notably, the previous study showed that neutralizing antibodies in convalescent plasma from peoples who have COVID-19 are less capable of recognizing a variant identified in South Africa, the 501Y.V2 VOC-(Alpha), than variants that circulated earlier in the pandemic [100].

Recent study showed that Alpha and Beta variants could partially escape humoral immunity induced by wild type SARS-CoV-2 infection or BNT162b2 vaccination, but not T-cell responses in COVID-19 convalescent donors and vaccinees [101]. In addition, early data suggest that immunity in convalescent individuals will be very long lasting and that convalescent individuals who receive available mRNA vaccines will produce antibodies and memory B cells that should be protective against circulating SARS-CoV-2 variants [102]. Furthermore, previous studies have shown that BNT162b2 and mRNA-1273 vaccines achieve only partial cross-neutralization of novel variants, supporting reformulation of existing vaccines to include diverse spike sequences [103].

In phase II/III vaccine efficacy studies conducted in the United Kingdom, ChAdOx1 nCoV-19 showed reduced neutralization activity against the B.1.1.7 variant compared with a non-B.1.1.7 variant in vitro, but the vaccine showed efficacy against the B.1.1.7 variant of SARS-CoV-2 (74.6%) [104]. In contrast, a multicenter, double-blind, randomized controlled trial showed that two doses of ChAdOx1 nCoV-19 vaccine did not confer protection against mild-to-moderate COVID-19 due to Beta variant [105]. However, recent data showed that two doses of ChAdOx1 nCoV-19 generated NAb activity against the wild type strain with a 2.1-fold reduction in median NAbT relative to two doses of BNT162b2. Moreover, medians of NAbTs against all SARS-CoV-2 variants were further reduced relative to BNT162b2, with 2.4-fold decrease against D614G and Alpha and 2.5-fold decrease against Beta and Delta variants [106].

In phase III trials, Ad26.COV2.S showed 52.0% vaccine efficacy after 28 days against moderate to severe-critical disease and 81.7% against severe-critical disease against Beta variant (20H/501Y.V2) [90]. In addition, recent findings suggest that the Beta variant does not escape the immunity induced by BBIBP-CorV [107].

A new strategy has emerged, called the ‘mix and match’ approach, for SARS-CoV-2 vaccination and may be required as proof against vaccine supply interruptions and also might help to reduce the transmission of emerging variants [108]. Indeed, preliminary data from a trial of participants received heterologous vaccination with the ChAdOx1 vector and either BNT162b2 or mRNA-1273 as booster dose induced stronger antibody responses than did the homologous ChAdOx1 vaccine series with acceptable reactogenicity profiles [109,110]. Interestingly, the ChAdOx1-BNT162b2 strategy results in 20- to >60-fold greater titers of NAbs against the Alpha, Beta, and Gamma variants [109].

## 5. Strategies for Monitoring of Humoral Immune Response during Infection or after Vaccination

### 5.1. Serological Assays

Serological tests measure the amount of antibodies produced against cognate antigens of the pathogen and are important for the identification of those who are immune [111]. Detection of SARS-CoV-2 Abs can be useful to confirm the presence of current or past infection [112,113].

Neutralization assays are used to measure NAb and require a biosafety level 3 laboratory (BSL-3), whereas serological tests might be cost effective and affordable for developing countries and do not require a BSL-3 facility [111]. Recently, several serological tests for the detection of SARS-CoV-2 antibodies have been developed and made available. Notably, many companies have rapidly developed tests for the detection of SARS-CoV-2 antibodies. These assays utilize various types of antigens and approved for IgG screening (Table 1). However, the major challenge for these assays is the absence of harmonization between different tests due to different antigens used in each immunoassay (Table 1).

### 5.2. SARS-CoV-2 Neutralization Assays

To better understand and characterize immunity to SARS-CoV-2 after natural infection or vaccination, there is a need for functional assays, such as virus neutralization assays, able to detect NAbs [114]. Serological assays are not designed to detect NAbs, which help to block interaction between virus and their receptors. The most relevant methods are the plaque reduction neutralization test (PRNT) and pseudotype virus assay.

The PRNT uses live virus incubated with dilutions of a patient’s sera or plasma in the BSL-3. The virus-serum mixtures are added onto Vero E6 cell monolayers and incubated 1 h at 37 °C in 5% CO_2_ incubator to determine if the sera inhibit or neutralize the cytopathological effect or plaque reduction is observed. Then, the plates are overlaid with agarose in cell culture medium and incubated for 3 days. The PRNT quantifies the neutralization titers associated with an individual’s clinical sample [114].

The reduction neutralization test uses a pseudotype virus, such as the vesicular stomatitis virus or lentivirus-based systems that incorporate SARS-CoV-2 S protein, and can be used in a biosafety level 2 laboratory [115]. The pseudovirus neutralization assay protocols are described in detail in several papers [116,117,118].

Recently, several alternative approaches using ELISA-based surrogate neutralization assays have been reported [119,120,121,122].

## 6. Conclusions

This review adds to the current understanding of humoral immune response following SARS-CoV-2 infection or after vaccination, which is critical for the prevention of secondary infections and vaccine efficacy. The IgM, IgA, and IgG Abs in individuals with COVID-19 disease were detected in the first week and second week, respectively, after symptom onset, whereas the dynamic of antibody response varies greatly among COVID-19 patients according to the age and the severity of disease.

Most participants receiving the COVID-19 vaccine developed specific humoral responses after second dose. In addition, evidence highlights that the immune responses induced by COVID-19 vaccination exceed those induced by natural SARS-CoV-2 infection, underscoring that people who have recovered from a COVID-19 infection still stand to benefit from vaccination [123]. However, several aspects of humoral immune response, threshold titers of neutralizing antibodies needed for protection, and the durability of the immunity induced by natural infection or after COVID-19 vaccine are still under investigation, and more specific conclusions about the protection against SARS-CoV-2 infection are expected to be drawn from future studies. Further investigations are needed to evaluate the maintenance of these immune responses and the potential need for booster doses. In this context, the potential of new variants to escape natural immune responses and vaccine-induced immunity makes the development of next-generation vaccines a high priority. Of course, as we currently have a platform for new technologies, such as nucleic acid-based or adenoviral vector vaccines, we will be able to quickly adapt more vaccines tailored to the emerging variants.

## Figures and Tables

**Table 1:** Approved SARS-CoV-2 IgG Immunoassays.

Company	Assay Name	Type	Antigen	Type of Test	Sensitivity (%)	Specificity (%)
Abbott Abbott Babson Diagnostics Beckman Coulter Beckman Coulter Roche Roche DiaSorin Inc. DiaSorin Inc. Beijing Wantai Beijing Wantai Beijing Wantai Euroimmun Euroimmun Euroimmun Bio-Rad Labs, Inc. BioMerieux Ortho-Clinical Diagnostics Ortho-Clinical Diagnostics Snibe Diagnostic Snibe Diagnostic Siemens Healthineers PerkinElmer Mindray Gold Standard Diagnostics Mediagnost	SARS-CoV-2 IgG SARS-CoV-2 IgG II Semi-Quant Babson Diagnostics aC19G1 Access SARS-CoV-2 IgG Access SARS-CoV-2 IgG II Elecsys Anti-SARS-CoV-2 Elecsys Anti-SARS-CoV-2 S Liaison SARS-CoV-2 S1/S2 IgG Liaison SARS-CoV-2 Trimeric S IgG SARS-CoV-2 Total Ab Wantai SARS-CoV-2 IgG (Quantitative) Wantai SARS-CoV-2 Neutralizing Abs Anti-SARS-CoV-2 IgG Anti-SARS-CoV-2 NCP Anti-SARS-CoV-2 QuantiVac Platelia SARS-CoV-2 Total Ab Vidas SARS-COV-2 IgG Vitros Anti-SARS-CoV-2 IgG Vitros Anti-SARS-CoV-2 Total Test Maglumi 2019-nCoV (SARS-CoV-2) IgM/IgG SARS-CoV-2 S-RBD IgG SARS-CoV-2 Total Assay GSP/DELFIA Anti-SARS-CoV-2 IgG SARS-CoV-2 IgG NovaLisa^®^ SARS-CoV-2 IgG Anti-SARS-CoV-2 IgG	CMIA ^1^ CMIA CLIA ^2^ CLIA CLIA ECLIA ^3^ ECLIA CLIA CLIA ELISA ^4^ ELISA ELISA ELISA ELISA ELISA ELISA ELFA ^5^ CLIA CLIA CLIA CLIA CLIA CLIA ELISA ELISA ELISA	N RBD ^?^ RBD RBD N RBD S1/S2 S RBD ^?^ - S1 N S1 N ^?^ S S ^?^ RBD S1 RBD S1 ^?^ S S1 RBD	Qualitative Semi-quantitative Qualitative Qualitative Semi-quantitative Qualitative Quantitative Quantitative Quantitative Qualitative Quantitative Quantitative Qualitative Qualitative Quantitative Semi-quantitative Qualitative Qualitative Qualitative Qualitative Qualitative Qualitative Qualitative Qualitative Qualitative Qualitative	100 100 100 96.8 100 100 98.8 97.9 98.7 94.4 100 - 90 94.6 90.3 97.5 96.6 90 100 89–95 100 100 96.2 ^?^ 99.9 95.3	99.9 99.9 100 99.6 98.9 99.8 100 98.5 99.5 100 100 - 100 99.8 99.8 99.6 99.9 100 100 96 99.6 99.8 100 ^?^ 99.5 98.6

^1^ Chemiluminescent microparticle immunoassay. ^2^ Chemiluminescent immunoassay. ^3^ Electro-chemiluminescence immunoassay. ^4^ Enzyme-linked immunosorbent assay. ^5^ Enzyme-linked fluorescent assay. ^?^ Not mentioned.

## Data Availability

Not applicable.

## Abbreviations

Ab	antibody
ACE2	angiotensin-converting enzyme 2
ARDS	acute respiratory distress syndrome
CLIA	chemiluminescent immunoassay
CMIA	chemiluminescent microparticle immunoassay
COVID-19	coronavirus disease 2019
E	envelope
ECLIA	electro-chemiluminescence immunoassay
ELFA	enzyme-linked fluorescent assay
ELISA	enzyme-linked immunosorbent assay
HCoVs	human coronaviruses
IgA	immunoglobulin A
IgG	immunoglobulin G
IgM	immunoglobulin M
M	membrane
MERS	Middle East respiratory syndrome
N	nucleocapsid
NAbs	neutralizing antibodies
PRNT	plaque reduction neutralization tests
RBD	receptor-binding domain
S	spike
SARS-CoV-2	severe acute respiratory syndrome coronavirus 2
SARS	severe acute respiratory syndrome
TMPRSS2	transmembrane protease serine 2
WHO	World Health Organization
